# Innate Antiviral Response: Role in HIV-1 Infection

**DOI:** 10.3390/v3071179

**Published:** 2011-07-14

**Authors:** Paula M. Pitha

**Affiliations:** 1 Department of Oncology, Sidney Kimmel Comprehensive Cancer Center, 401 North Broadway, Baltimore, MD 21231, USA; 2 Department of Molecular Biology and Genetics, Johns Hopkins School of Medicine, 725 North Wolfe Street, Baltimore, MD 21205, USA; 3 Department of Biology, Johns Hopkins University, 3400 N Charles St. Baltimore, MD 21218, USA; E-Mail: parowe@jhmi.edu; Tel.: +1-410-516-6954; Fax: +1-410-516-5213

**Keywords:** virus, HIV-1, interferon, IRF, innate immune response

## Abstract

As an early response to infection, cells induce a profile of the early inflammatory proteins including antiviral cytokines and chemokines. Two families of transcriptional factors play a major role in the transcriptional activation of the early inflammatory genes: The well-characterized family of NFkB factors and the family of interferon regulatory factors (IRF). The IRFs play a critical role in the induction of type I interferon (IFN) and chemokine genes, as well as genes mediating antiviral, antibacterial, and inflammatory responses. Type I IFNs represent critical components of innate antiviral immunity. These proteins not only exert direct antiviral effects, but also induce maturation of dendritic cells (DC), and enhance functions of NK, T and B cells, and macrophages. This review will summarize the current knowledge of the mechanisms leading to the innate antiviral response with a focus on its role in the regulation of HIV-1 infection and pathogenicity. We would like this review to be both historical and a future perspective.

## 1. Introduction

The ability of the host to detect invasion by a pathogenic intruder and to activate defense mechanisms to eliminate infection is essential for survival. The innate immune response has developed as a rapid and regulated defense mechanism against invading pathogens. Remarkable progress has been made in recent years into the identification of cellular receptors detecting the invading pathogens as well as in understanding the signaling pathways leading to the induction of Type I IFN genes and inflammatory cytokines. It has been shown that the recognition of an invading pathogenic organism can occur upon binding of viral nucleic acids or envelope proteins to specific membrane associate receptors, denoted Toll-like (TLR) receptors, which can recognize conserved patterns of proteins, lipoproteins, and viral RNA [1]. In addition, cytoplasmic sensors can recognize viral RNA or DNA in the B configuration of the invading pathogens. In addition, the cellular polymerase III that converts cytoplasmic DNA into 5′phosphorylated RNA recognized by RIGI is also an important component of the innate virus detection system. The binding of viral ligands to host sensors induces multiple signaling pathways that activate cellular transcription factors controlling the expression of a diverse set of genes, which in turn coordinate both the innate and adaptive immune responses. Although cascades of multiple kinases and ubiquitination steps usually mediate activation of these transcription factors, their functional diversity is modulated by interaction with other transcription factors and cofactors. Indeed, these regulatory networks are critical components of the host defense against invading pathogens, including viruses.

In response to infection, cells induce early inflammatory proteins including type I IFN and inflammatory cytokines. Two families of transcriptional factors play a major role in the stimulation of expression of these proteins: the well-characterized family of NFkB factors and the family of interferon regulatory factors (IRF). The IRFs play a critical role in the induction of Type I interferon (IFN) and chemokines as well as proteins mediating antiviral, antibacterial, and inflammatory responses [2,3]. Type I IFNs are critical components of the innate antiviral response. These proteins not only exert direct antiviral effects, but also induce maturation of dendritic cells (DC), recruitment of the immune cells to the sites of infection, and enhance the functions of macrophages, NK, T and B cells, and macrophages [4]. The importance of Type I IFN to the activation of effector cell populations and adaptive immunity is also emerging. This chapter will review the current knowledge of the molecular mechanisms leading to the innate antiviral response with a focus on its role in HIV-1 infection. We would like this review to have both a historical and a future perspective.

## 2. Transcription Factors of the IRF Family

The IRFs are transcriptional mediators of virus, bacteria and IFN-induced signaling pathways and as such play a critical role in antiviral defense, immune response, cell growth regulation and apoptosis. To date, nine human cellular IRF genes (IRF-1, IRF-2, IRF-3, IRF-4/Pip/ICSAT, IRF-5, IRF-6, IRF-7, ICSBP/IRF-8 and ISGF3γ/p48/IRF-9), as well as virus-encoded analogues of cellular IRFs have been identified [5,6]. These factors can function as transcriptional activators (e.g., IRF-1, IRF-3, and IRF-9), repressors (e.g., IRF-8) or both (e.g., IRF-2, IRF-4, IRF-5). They all share significant homology in the N-terminal 115 amino acids, which comprise the DNA-binding domain, characterized by five tryptophan repeats. Three of these repeats contact DNA with specific recognition of 5′GAAA3′ and 5′AANNGAAA3′ sequences [7]. However, the unique function of a particular IRF is accounted for by cell type-specific expression, intrinsic transactivation potential, and an ability to interact with other members of the IRF family or other transcription factors and co-factors [8]. All IRFs, except IRF-1 and IRF-2, contain the IRF-associated domain (IAD) in the 3′ terminal part of the protein, which mediates these interactions.

The first IRFs, IRF-1 and IRF-2, were identified through their ability to bind to the virus responsive element (VRE) of the *IfnB* gene and were proposed to function as an activator and repressor of the *IfnB* gene, respectively [9]. However, homozygous deletion of IRF-1 in mice did not impair activation of *IfnA* or *IfnB* genes in infected mouse embryo fibroblasts (MEFs), while dsRNA-mediated induction of Type I IFN was down-regulated [10]. Subsequent studies have revealed that IRF-1 is involved in a broad spectrum of antiviral defenses mediated by IFN-γ by activation of the *IfnG* genes. Furthermore, analysis of the repertoire of lymphoid cells from mice devoid of IRF-1(*Irf*1 null) has shown defects in the maturation of CD8+ T cells as well as a defective Th1 response, impaired production of IL-12 in macrophages, and defective NK cell development.

Although IRF-1 stimulates expression of the *IfnG* gene, it does not have a critical role in viral mediated stimulation of Type I *IfnB* genes. However, subsequent identification of three IRFs (IRF-3, IRF-5 and IRF-7) showed that they are direct transducers of virus-mediated signaling and demonstrated their crucial role in the expression of Type I *Ifn* genes and some chemokines [6,11–13]. The identification of IRF-3 and IRF-7 and their role in the transcriptional activation of type I *Ifn* genes had a major impact on the understanding of the molecular mechanism of the pathogen-induced innate antiviral response [14–17]. In human cells, multiple spliced variants of these IRFs can be detected, and some of these variants can function as dominant negative mutants. In infected cells, the ubiquitously expressed IRF-3 mediates induction of IFNβ and of some IFN induced genes (ISG), whereas expression of IRF-5 and IRF-7 is limited largely to lymphoid cells, where they are required for the expression of the *IfnA* genes [3,14]. Reconstitution of IRF-5 or IRF-7 expression in infected fibroblasts that express only IFNβ confirmed expression of IFNα subtypes [18]. In many cells, IRF-3 and IRF-7 are involved in the amplification of the interferon response: antiviral response is generally induced through two sequential steps: (1) virus activates IRF-3, which leads to synthesis of IFNβ. (2) IFNβ stimulates transcription of IRF-7, which results in synthesis of IFNα and further enhancement of IFNβ synthesis [12].

High constitutive levels of both IRF-5 and IRF-7 were detected in plasmacytoid dendritic cells (PDC), which are high IFNα producers [19,20]. Subtypes of *IfnA* genes induced by IRF-5 and IRF-7 in B cells are distinct, indicating that these two factors have both essential and non-redundant functions [21]. IRF-7 expression is critical for induction of *IfnA* genes both *in vitro and in vivo.* Virus infection or CpG DNA was not able to stimulate expression of Type I *Ifn* genes in *Irf7−/−* mice. The authors concluded, therefore, that IRF-7 is the master regulator of type I IFN [22], although residual IFN production, mediated by IRF-3, could still be induced in cells of non-lymphoid origin. In contrast *Irf5−/−* mice showed not only a decrease in virus mediated induction of Type I IFN, but also a significant decrease in expression of inflammatory cytokines such as TNFα, IL-6 and IL-12 [23,24]. Furthermore, recent data indicate that IRF-5 has a critical role in the development of TH-1 responses to *Leishmania donovani* infection [25] as well as in the differentiation and function of B cells [26] and macrophages [27]. Thus, while the role of IRF-7 is critical for the innate antiviral response, IRF-5 function is broader, and can mediate the cross-talk between innate and adaptive immune responses.

### 2.1. The Viral IRF: KSHV-Encoded Viral IRF

Kaposi’s sarcoma-associated herpes virus (KSHV) is a member of the γ herpes virus family and is genetically similar to EBV and monkey Herpes Virus Saimiri (HVS) [28]. Sequence analysis of the KSHV genome revealed the presence of about 80 open reading frames (ORFs) and a number of ORFs showing homology to cellular genes including a cluster of four ORFs with homology to the cellular IRF family transcription factors [29], three of which have been cloned and characterized. The open reading frame 9 of KSHV genome (ORF K9)-encoded vIRF-1, has been studied most extensively and was shown to inhibit both virus-mediated induction of Type I *Ifn* genes and IFN-induced genes (*ISG*) [30–34].

vIRF-2 (ORF K11.1) encodes a small nuclear protein (163 aa) that unlike the cellular IRFs, binds dsRNA-activated protein kinase (PKR), inhibits its kinase activity and blocks the phosphorylation of the PKR substrate, eukaryotic translation initiation factor 2α [34,35].

vIRF-3/LANA2, encoded by ORFs K10.5 and K10.6 [36,37], is a multifunctional nuclear protein constitutively expressed in KSHV-positive B cell lymphoma cells (PEL) and Castleman’s disease tumors [38]. The vIRF-3 protein binds to IRF-3, IRF-7 and IRF-5 [37,38] vIRF-3 also interacts with two tumor suppressor genes, p53 and MM-1 [39].

### 2.2. IRF Mediated Cross Talk between Innate and Adaptive Immune Response

The observation that some IRFs also have an essential role in the differentiation and function of lymphoid cells emerged mainly from a large number of studies in genetically modified mice. Thus, IRF-1 participates not only in the induction of the *IfnG* gene, but also in several autocrine loops that induce and enhance the Th1 response [40,41]. IRF-4 has been also associated with Th cell development and the differentiation of B cells to plasma cells [42]; *Irf4−/−* mice show a complete absence of plasma cells [43]. *Irf8−/−* mice show major defects in CD8+DC and plasmacytoid dendritic cells (PDC). These mice also display increased susceptibility to infection, which is due to a defect in the Th1 immune response and an inability to express IL-12 [44,45]. IRF-3 and IRF-7 play essential roles in the antiviral response, however IRF-7 also has an important role in the differentiation of monocytes to macrophages [46]. The Th1 and Th2 responses of *Irf3−/−* or *Irf7−/−* mice have yet to be characterized, however a DNA-mediated Th1 immune response was enhanced by co-expression of IRF-3 and IRF-7 [47]. IRF-5 plays an important role in B cell differentiation and function *in vitro.* Infected B cells over expressing IRF-5 show up-regulated expression of several immune response genes, including CD80, MyD88 and ISG [21], and IRF-5 promotes macrophage polarization and TH-1 response [25,27]. *Irf5−/− mice* show a defect in differentiation of B cells to plasma cells and an attenuated response to antigenic stimulation [26]. IRF-9 is an important component of the ISGF-3 complex with an essential role in the type I IFN signaling pathway and induction of many ISGs including IRF-7 and IRF-5 [48]. The *Ifr9−/−* mice show a significant defect in Type I IFN signaling and share many characteristics with the *Stat1−/−* and *In*fR1*−/−* mice. These mice are also predicted to be defective in expression of many ISGs including both IRF-7 and IRF-5. However, whether they show modulation of the adaptive immune response similar to *Irf5−/−* mice has yet to be evaluated.

## 3. Induction of the Antiviral Response

In has been shown recently that both membrane bound receptors and cytoplasmic cellular sensors recognize viral infection. Although the signaling pathways induced by these receptors/sensors are distinct, they generally result in activation of two members of the IRF family, IRF-3 and IRF-7. The majority of uninfected cells express IRF-3 constitutively, which is present predominantly in the cytoplasm. Upon virus-induced phosphorylation at the carboxyl terminal serines, IRF-3 translocates to the nucleus, where it forms a transcriptional complex—the enhanceosome—with the transcription co-activator p300/CBP, NfkB and Jun, which subsequently binds to interferon responsive elements (IRF-E) in the promoters of *IfnB* gene [49]. IRF-7 can be activated by a similar pathway and is a member of a multicomponent complex, binding to the promoters of *IfnA* genes [13,50,51]. IRF-5 shows structural and functional features distinct from IRF-3 and IRF-7. The IRF-5 polypeptide contains two nuclear localization signals that are not present in IRF-3 or IRF-7, and low levels of nuclear IRF-5 can be detected in uninfected cells. Activation of IRF-5 appears to be virus-specific, and it can be phosphorylated in NDV- or VSV-infected but not in Sendai virus-infected cells or in poly IC treated cells [6].

### 3.1. The Role of IRF in Toll Receptor Mediated Antiviral Pathway

Understanding the mechanism by which cells detect invading pathogens was significantly advanced by the observation that this immune response is initiated by Toll like receptors (TLR) [52,53]. The TLR are Type I transmembrane protein receptors expressed in endosomes that recognize conserved motifs unique to pathogens, denoted as pathogen-associated molecular patterns (PAMPS). The presence of dsRNA, which has been considered the common signature of virus infected cells, is recognized by TLR3, while ssRNA activates TLR7/8, and unmethylated dsDNA activates TLR9. The binding of viral nucleic acids to their respective TLR induces signaling pathways leading to the activation of IRF and NFkB. Some TLR, like TLR2 can form homo or heterodimers, which alters the specificity of the ligand binding. With the exception of TLR3, activation of all TLR family members is dependent on the adaptor MyD88 [17]. Upon ligand activation, the intracellular part of TLR-TIR binds a tetramer of MyD88, and the signaling pathway is initiated by recruitment of IRAK1 and IRAK4, which are autophosphorylated and form a complex with the E3 ubiqitin ligase TRAF6 [54]. This can activate mitogen activated protein kinase and lead to the activation of NFkB, which binds to the promoters of inflammatory genes such as IL-6 and TNFα or leads to the activation of IRF [17,55].

The expression of TLR is cell type specific. Thus the PDC, which represent the major source of Type I IFN, (largely IFNα), constitutively express TLR7/8 and TLR9 as well as relatively high levels of IRF-5 and IRF-7 [56]. In contrast, TLR3 signaling is distinct, it does not associate with MyD88, and binding of dsRNA to the TLR3 receptor activates IRF-3 through the TRIF adaptor [57]. Two non-canonical IKB kinases, IKKε and TBK-1, have been implicated in the phosphorylation and activation of IRF-3 and IRF-7 in cultured human cell lines *in vitro* [58]. Activated IRF-3 dimerizes and binds to p300/CBP co activators [49], translocates to the nucleus and activates transcription of the *IfnB* gene and some *ISGs* in IFN-independent manners [59]. Both IKKε and TBK-1 kinases also phosphorylate IKBα, but only on serine 36, which does not lead to the degradation of IKBα by the ubiquitination pathway or to activation of NFkB [60].

The TLR adaptor, TRIF, is a potent activator of the *IfnB* promoter. Mice that are *Trif*-deficient or have a mutation in the *Trif* gene [61] have a profound defect in IRF-3 activation and fail to produce IFNβ. In addition, activation of IRF-3 and stimulation of *IfnB* and *Rantes* in infected or LPS-treated cells does not occur in TBK-1-deficient cells. It is noteworthy that whereas TRIF was shown to be solely responsible for the TLR3-mediated activation of IRF-3, activation by TLR4 requires an additional adaptor protein MAL [62]. Taken together, these results indicate that the initial signaling events leading to the activation of TBK-1 by TLR3 and TLR4 may not be identical. However, there is strong evidence that IRF-3 plays an essential role in the antiviral response. First, ubiquitous expression in most of the cell types, allows stimulation of the antiviral response and synthesis of IFNβ in a variety of infected cell types [14]. Second, even low levels of autocrine or paracrine IFNβ stimulate expression of *Irf7* and *Irf5* and triggers amplification of the antiviral response [12,63]. Finally, the observation that many viruses prevent the induction of Type I IFN by targeting the function of IRF-3, underlines a central role for IRF-3 in the induction of the antiviral response [64].

TLR7/8 and TLR9 are an evolutionarily related subgroup within the TLR family [65]. Both of these receptors are expressed in PDC, and act intracellularly in association with endosomes in a MyD88 dependent fashion. The TLR7/8 and TLR9 signaling pathway activates IRF-7 and IRF-5, (but not IRF-3). While the unmethylated CpG DNA [66] specifically stimulates TLR9, single stranded (ss) viral RNA and synthetic siRNA are recognized by TLR7 and TLR8 [67]. In mice, TLR7 is activated by infections with ssRNA viruses, including influenza virus and vesicular stomatitis virus (VSV) [67,68]. The activation of MyD88 pathways is initiated by the assembly of a multicomponent complex containing MyD88 tetramers, IRAK1 and IRAK4, TRAF6 and IRF-7 and or IRF-5. Activation of these two IRFs requires ubiqiutination by TRAF6 and phosphorylation by a not yet well defined kinase. Thus while IRF-7 can be activated both by TRIF and MyD88 pathways, IRF-5 is activated only by TLR7 and TLR9, and the activation is dependent on MyD88 and K63 ubiquitination by TRAF6 [69,70].

### 3.2. The Role of CARD-containing Proteins in Activation of IRF-3

dsRNA, generated as an intermediate during virus replication, can be also recognized by cytoplasmic sensors. Two closely related intracellular RNA helicases, Rig-I and MDA5, which are ubiquitously expressed in nearly all cell types, can recognize dsRNA and 5′triphosphorylated RNA. Both helicases contain two N’ terminal caspase-recruiting domains (CARD) that activate downstream signaling pathways and a C-terminal DExD/H helicase domain that binds RNA. Signaling by Rig-I is mediated through downstream interaction with the adaptor protein IPS-1/MAVS. Both sensors contain CARD domains, and conformational changes occurring upon RNA binding permit these domains to interact with the CARD domain of an adaptor IPS-1/MA*VS.* This protein is localized on the outer mitochondrial membrane and this association is critical for its activity. IPS-1 activates TBK-1 and IKKe that leads to the activation of IRF-3, IRF-7 and NFkB. The IPS-1-dependent signaling is an essential feature of host immunity to RNA virus infection [71,72].

### 3.3. Recognition of DNA Viruses

The analysis of TLR ligands clearly shows that the endosomal TLR9 senses hypo/unmethylated CpG DNA that is present in genomes of bacteria and viruses. TLR9 is also the major sensing mechanism for DNA viruses and TLR9 dependent recognition of virus infection results in production of Type I IFN [73]. Rather unexpected was the observation that TLR2 plays a critical role in recognition of herpes viruses. In DC that expresses multiple TLR receptors, HSV-2 engaged both TLR2 and TLR9, but the TLR2 recognition was limited only to a distinct population of HSV-2 [74]. The synergistic interaction between TLR2 and TLR9 was also found to control HSV-2 infection in brain [75]. It has been assumed that TLR2 detects viral envelope glycoprotein [76].

As shown later, detection of viral DNA by cytoplasmic DNA sensors also leads to a profound antiviral response, and multiple cytoplasmic DNA sensors have been identified [77]. A common feature of the signaling pathway induced by these receptors is activation of a transmembrane protein present on the endoplasmic reticulum designated STING (stimulator of IFN genes), which activates TBK-1 kinase that phosphorylates and activates IRF-3 and IRF-7 [78]. Redundancy may be another feature of these sensors. Thus DAI (DNA dependent activator of IRF) induces *in vitro* activation of Type I IFN in response to synthetic DNA and HSV infection by the TBK-1-IRF-3 axis, but DAI knockout mice did not show any attenuation of IFN induction [79,80]. Another DNA sensor, interferon induced protein 16 (IF16) also interacts with STING and activates the TBK-1-IRF-3 axis and Type I IFN production [81]. Two cytoplasmic sensors recognize AT-rich B form DNA. The first sensor is leucine rich repeat Fli-I interacting protein (LRRFIPI) that activates IFN production by association with β Catenin, followed by recruitment of p300 histone acetyltransferase and IRF-3 to the *IfnB* promoter [77]. The second sensor is RNA polymerase III, which recognizes AT rich DNA and transcribes it to immunostimulatory RNA recognized by Rig I [82]. The importance of these two sensors for sensing viral infection remains poorly characterized.

The response to DNA in the cytoplasm can also be mediated through inflammasome complexes, resulting in the production of inflammatory cytokines that are also an important part of the innate immune response [83]. Among these mediators is the protein AIM2 (absent in melanoma), which is an interferon induced protein that recognizes viral DNA and triggers the assembly of the inflammasome complex, resulting in activation of inflammatory cytokines IL1β and IL18, but not Type I IFN [77].

## 4. Interferon-Stimulated Genes: Mediators of the Antiviral Effects

The Type I IFN system has a critical role in the control of viral infection. Defects in the IFN system, in the genetically modified mice lacking functional type I IFN receptors and in humans with genetic defects in the type I IFN induction or response, result in inability to control viral infection [84,85] even in the presence of fully functional adaptive immune responses [86]. The antiviral effect of IFN is mediated by the induction of a large number of cellular genes (*ISG*) [87], which encode proteins with diverse functions including antiviral properties, pro-apoptotic functions and modulation of ubiquitination pathways [88]. Induction of these genes is initiated upon binding of Type I IFN to its cellular receptor, which initiates receptor-mediated signaling pathways involving the activation of two receptor associated kinases (JAK1 and Tyk2), and the consequent tyrosine phosphorylation of pre-existing signal transducers and activators of transcription (STAT) [89]. Upon phosphorylation, STAT1 and STAT2 assemble together with interferon responsive factor 9 (IRF-9) into a multimeric complex (ISGF3) [90], which interacts with interferon-responsive elements (ISRE) present in the 5′ flanking region of *ISG* and activates their transcription [87,91]. Type I IFN also stimulates the formation of STAT1 homodimers, which bind to the IFN-γ-activated site (GAS), present in the *ISG* promoters that can be induced both by Type I IFN and IFNγ [92]. In addition, the STAT2-IRF-9 heterodimer is also an activator of *ISG* transcription. Signaling by Type I IFN also can activate both the MAPK and PI3K pathways [93], although the contribution of these two pathways to the antiviral response *in vivo* is not fully established.

The antiviral function of the majority of ISG has yet to be determined. Several of the interferon-induced antiviral pathways have been described, and the component proteins have been identified [94,95]. The earliest characterized ISG, 2′,5′-OAS, induces a pathway that leads to viral RNA degradation. Low levels of constitutive 2′,5′-OAS are up regulated by Type I IFN and when activated by dsRNA, the 2′,5′-OAS enzyme synthesizes pppA(2′p5′A)n, (2′,5′A oligoadenylates), which activate the cellular endonuclease, RNase L. Binding of 2′5′A oligoadenylates to RNAse L cleaves both cellular and viral RNAs at UU or AU nucleotides [96].

Constitutively expressed RNA dependent protein kinase (PKR) is also induced by Type I IFN. The inactive monomers are activated by binding viral RNA; PKR is subsequently phosphorylated and forms active dimers. Activated PKR dimers catalyze phosphorylation of several substrates including the α subunit of the initiation factor eIF-2 (eIF-2α) [97], as well as the transcription factor inhibitor IκB [98].

The Mx proteins are GTPases, which are induced by IFNα/β but not by IFNγ [99]. The Mx proteins accumulate in the cytoplasm or endoplasmic reticulum, where they are present as oligomers. In infected cells, Mx monomers are released to the cytoplasm and bind essential viral components and then degrade them. Mx proteins confer a high degree of antiviral resistance to infection for large groups of viruses [100]. The role of the OAS, PKR, and Mx pathways in resistance to viral infection was clearly established in genetic studies; mice that are lacking any of these pathways have decreased resistance to viral infection. Surprisingly however, some antiviral resistance still remained in mice in which all three pathways were deleted indicating that not all the cell mediated resistance to virus infection is mediated by these three pathways [95,101].

Several additional ISG were shown to have antiviral functions. ISG15, one of the first identified ISG, is a ubiquitin-like protein that can be covalently attached to the lysine residues of viral and cellular proteins, and thereby direct interplay between ubiquitination and ISGylation [102]. Many of the ISG 15 targeted proteins have an important role in the interferon system. Unlike ubiquitination, ISGylation does not target proteins for degradation but can prevent Ub mediated degradation. Thus ISG15 was show to interfere with the ubiquitination pathway essential for assembly and release of HIV-1 and Ebola virus virions [103,104] (see below).

Type I IFN induced endonuclease ISG20, is specific for ssRNA. Furthermore, IFN induces two nucleic acid editing enzymes, adenosine deaminase (ADAR) acting on RNA and APOBEC3G. ADAR-mediates transition of nucleotides from A to I, which disrupts base pairing and the AU base pair is replaced by the less stable IU pair that destabilizes dsRNA. This A-I editing has been found in multiple viral RNA sites of negative strand RNA viruses, and has been associated with persistent infection [105]. APOBEC3G is a cytosine deaminase that converts cytidine to uridine in single stranded proviral DNA, which results in hyper mutation of the HIV-1 genome. Expression of APOBEC3G has been shown to be up-regulated by IFNα [106,107]. Thus both APOBEC3G and ADAR are IFN-induced antiviral proteins, which can induce hyper mutation of the viral genome and decrease viral fitness.

Finally, the role of IFN-induced miRNA in the antiviral response has been reported recently and their number and importance has been given high attention [108,109].

During the early days of IFN research, it was assumed that the interferon-mediated inhibition of viral replication was caused by a common mechanism affecting a large number of different viruses. Instead, it has become clear that the antiviral effect is due to the combinatory effects of many proteins, and any antiviral protein may show specificity for a distinct group of viruses.

## 5. Viral Strategy to Overcome the Antiviral Response

The effective antiviral response limits the ability of a virus to replicate. To overcome the cell mediated block, most viruses developed mechanisms to eliminate the interferon function by acquiring genes encoding nonstructural proteins that are non-essential for viral replication but can block the antiviral IFN response. Since the capacity of viral genomes is limited, many of the viral antagonists simultaneously inhibit multiple components of the IFN system, thus achieving inhibition of the antiviral response by a limited number of proteins. Thus a single viral protein like paramyxovirus V protein targets both the critical components in the signaling pathways leading to both type I IFN induction and IFN action [110,111]. The common strategies are the inhibition of IFN induction by targeting steps involved in IRF-3 activation [35,112] or promoting its degradation [113] and inhibition of Type I IFN signaling pathways by activation of the cellular suppressors of STAT, SOCS-1 and 3 [114]. The large DNA viruses express several proteins that can inhibit the antiviral response using alternative approaches. Thus KSHV expresses several analogues of cellular IRF, vIRF1-4 that have dominant negative effects on cellular IRF [30,33,34,36]. Alternatively pox viruses encode soluble IFN receptors that compete with IFN for binding to cellular receptors [115].

Another viral approach to circumvent innate immunity, used preferentially by lytic viruses, is to inhibit a basic cellular mechanism such as synthesis of host RNA and proteins [116]. Taken together, these data indicate that the ability of the virus to establish a spreading infection depends on a well-controlled balance between the initial cellular antiviral response and the ability of the virus to overcome it.

## 6. Role of the Innate Immune Response in HIV-1 Infection

It has been well established that HIV-1 infection is associated with the modulation and activation of the immune system [117], characterized by a progressive decline and dysfunction of T cells, impaired function of NK cells, and polyclonal activation of B cells. The levels of immune activation are an indication of the progression of the HIV-1 disease. When the cytokine response induced during the early stages of HIV-1 was evaluated by the analysis of cytokine plasma levels or cytokine expression in PBMC, in most cases there was up-regulation of cytokines and chemokines including IFN, IL-1 and TNFα and decreased levels of IL-6 and IL-2. Furthermore, the expression of Type I IFNβ was transient and two consecutive peaks in cytokine levels were observed. The first peak, which occurred at about 6 days after the start of viremia, was transient and showed an increase in IFN-α, IL-15, TNF-α, IP-10, and MCP-1, and the second peak, associated with production of IL-6, IL-8, IL-10, IL-18, and IFN-γ, was detected 7 to 12 days post infection [118]. This data indicate that the early steps of HIV1 infection are associated with activation of the innate antiviral and inflammatory response prior to development of the adaptive immune response. Potential cross talk between innate and adaptive immunity in HIV-1 pathogenicity and its mechanism has not been investigated in detail. The profound innate inflammatory response to HIV-1 infection leading to the induction of an antiviral response may also have detrimental effects. The expression of pro-inflammatory cytokines may promote HIV-1 replication and suppress the antiviral effect of Type I IFN. Stimulation of HIV-1 replication by TNFα and IL-6 was shown *in vitro* and may also occur *in vivo* [119]. Another factor of the innate immune system, IRF-1, was recently implicated in the regulation of HIV-1 infection. IRF-1 is expressed constitutively at low levels, but its expression is strongly up-regulated by IFN γ. IRF-1 was also induced in T cells during early steps of HIV-1 infection [120] and the expression of IRF-1 was also observed in non-permissive HIV-1 infection of DC. In these cells, HIV-1 induced increased expression of IRF-1 and IRF-7, but the activation of IRF-3 was suppressed [121]. The 5′LTR region of HIV-1 contains an IRF-1 binding site, and its deletion impairs HIV-1 replication *in vitro* [122]. Interestingly, specific IRF1 polymorphisms have been found in a small group of people showing resistance to HIV-1 infection and pathogenicity (non progressors). This polymorphism was associated with the reduced levels of IRF-1 in PBMC, suggesting that the decrease in IRF1 expression may limit the susceptibility to HIV infection as IRF-1 contributes to stimulation of HIV-1 replication [123].

The detrimental role for immune activation in HIV infection is supported by animal studies. In the pathogenic SIV animal model of HIV, induction of the strong innate inflammatory response is characterized by production of Type I IFN during the very early stages of SIV infection; in contrast, in nonpathogenic SIV infection the induction of inflammation and Type I IFN is only transient [124].

### 6.1. HIV-1 Infection *in vitro* Induces Interferon Signature

*In vitro*, HIV-1 infects a number of diverse cell types; however the characteristics of the infections in these cells are distinct. Thus while infection in activated CD4+ T cells is productive and cytopathic, in naïve T cells HIV-1 is present in latent state. In macrophages HIV-1 replicates at low levels and does not induce cytopathic effects. Thus the innate antiviral response to HIV-1 infection in different cell types may be distinct. Several cell types, including CD4+ T cells, primary macrophages, and PBMC, infected with HIV-1 *in vitro* induced interferon signature, while induction of either IFNα or IFNβ was generally difficult to detect. Analysis of this typical interferon signature transcription profile in HIV infected CD4+T cells showed significant up-regulation of expression of interferon stimulated genes with increasing viral load, including genes of the intrinsic antiretroviral defense. Upon successful antiretroviral treatment, the signature profile of previously viremic individuals reverted to a pattern comparable to that of non progressors and uninfected individuals [125]. In contrast, when the transcription profile of lymph nodes from HIV-1 infected patients was analyzed, only about 5% of the identified genes were associated with the antiviral response and the majority of the genes were negatively associated with HIV-1 replication. Based on these data the authors concluded that the antiviral response is not sufficient to attenuate HIV-1 replication [126]. Recent data have shown up-regulation of type I IFN induced ISG in activated HIV-1+CD4+ T cells [127], indicating that only a subset of CD4+ cells can induce IFNα. However the IFN signature induced in PBMC can be also induced by exogenous Type I IFN produced by other types of cells such as PDC, which are high producers of IFNα. The interaction between HIV-1 and DC is complex and not yet fully understood. It was shown that PDC are recruited to the site of HIV-1 replication in HIV-positive lymph nodes and this recruitment has been facilitated by expression of increased levels of lymphocyte homing marker CCR7 on circulating DC of HIV-1 infected individuals [128]. Activated DC from HIV-1 infected individuals expressed high levels of IFNα and other inflammatory cytokines and these could well contribute to increased HIV-1 replication through immune activation.

The mechanism and the signaling pathway by which HIV-1 activates IFN production in dendritic cells has been subject of intensive studies. The PDC are an essential part of the innate immune system and the major producer of IFNα It was shown that the co-culture of PBMC with HIV-1 infected lymphocytes or infected CD4+ cells resulted in production of relative high levels of Type I IFN and that the major producers were the DC [118]. Production of IFNα was observed in lymph nodes of AIDS patients [128] and has been associated with disease progression [129,130]. Recent clinical data suggest that production of IFNα may drive the hyper activation of the immune system resulting in CD4 T cell depletion and AIDS [127]. Presently, it is assumed that IFNα alone contributes to the progression of AIDS disease, however a decrease in CD4+T cells is also associated with a significant increase in serum levels of inflammatory cytokines including TNFα. IL-6, IL-18 and IL-7 which may contribute to the observed hyper activation of the immune system.

The DC are generally resistant to HIV-1 infection, and co-culture of HIV-1 particles with monocyte derived dendritic cells (MDDC) neither infects DC or stimulates their activation. However when the freshly isolated DCs were infected with various HIV-1 strains *in vivo*, they were permissive for productive infection with the macrophage tropic HIV-1 (R5 HIV-1) and the infected DCs transferred HIV-1 to CD4 T cells [131]. It was shown that DC and T cells can form conjugates that facilitate transfer of HIV-1 from DC to T cells [132]. It was also shown that this transfer is bi-phasic [133].

As described above, cell recognize RNA viruses either by the membrane bound receptors such as TOLL receptors or cytoplasmic receptors such as RNA helicase RigI and MDA5. Which of these receptors are engaged upon HIV-1 infection has not been fully explored. However it was shown that the peptide inhibitors of TLR7 ligands inhibited IFN production in PBMC co-cultivated with HIV-1 infected T cells, indicating that the HIV-1 recognition is at least partially mediated by TLR7 [130,134]. The TLR7 independent pathway, which depends on IRF-3, was also induced in the co-culture experiments of HIV-1 infected T cells with TLR negative cells. Interestingly the induction of Type I IFN did not depend on HIV-1 replication in the infected cells, but was facilitated by the fusion of the infected T cells with DC [134]. It is possible that the fusion transfers large amounts of viral nucleic acids to the cytoplasm of DC, where the viral RNA may be recognized either by TLR7 or one of the cytoplasmic sensors such as Rig I or MDA5. Alternatively, the un-integrated proviral DNA could be recognized by one of the recently identified cytoplasmic DNA sensors. Further studies will certainly address these questions.

Another very interesting and novel aspect of the mechanism of IFNα induction in DC is the observation that the restriction of HIV-1 replication in DC can be overcome by the SIV encoded protein Vpx. Expression of this protein or co-infection with SIV (that encodes Vpx) allowed HIV-1 replication in DC, stimulated production of IFNα and activated DC [135]. It was also shown recently that Vpx induces proteasomal degradation of SAMHD1, a negative regulator of the interferon response. Silencing of SAMHD1 in non-permissive cell lines alleviated HIV-1 restriction and overexpression of SAMHD1 in sensitive cells attenuated HIV-1 replication [136]. Further experiments demonstrated requirement of an interaction of HIV-1 capsid with CYPA for IFN production [135]. The induction of IFN was also dependent on nuclear localization of phosphorylated IRF-3. Whether the phosphorylation and activation of IRF-3 in these cells depends entirely on the capsid interaction with cellular proteins or whether the requirement for the capsid interaction is connected to the TLR3 or RIG I-IRF-3 axis needs yet to be clarified. However it is unlikely that the SAMHD1 is the only factor that can modulate HIV-1 replication in DC. The attenuation of HIV-1 replication by the intrinsic cellular antiviral proteins such as APOBECC3G, TRIM and others [95] may also contribute to this inhibition.

### 6.2. Factors Counteracting the Antiviral Response to HIV-1 Infection

In addition to the antiviral ISG described earlier, type I IFN induces expression of several ISGs that interfere with different stages of HIV-1 and retroviral replication. These proteins are constitutively expressed at low levels in a cells type specific manner, and unlike the antiviral enzymes PKR or 2′5′OAS (already described above), they do not seem to require virus mediated activation (reviewed in [137,138]. The first of these cellular factors, dubbed intrinsic immunity factors was APOBEC3G [139]. APOBEC3G is a cytidine deaminase that represses reverse transcription of viral RNA, acts on CC dinucleotide motifs and induces deamination of cytidine to uridine during synthesis of negative strand proviral DNA transcripts. This change leads to a G to A transition in proviral DNA and decreased ability (fitness) in virus replication. The expression of APOBEC3G is stimulated by Type I IFN [140], and depends on NFATc1/NFATc2 and IRF-4. When either NFATc1 or NFATc2 and IRF-4 were co-expressed, activity of the APOBEC3G promoter was observed in cells that normally lack APOBEC3G expression. In addition to its effect on virus fitness APOBEC3G editing results in misfolding of viral proteins and enhances their recognition by CTL cells, thereby stimulating the antiviral adaptive immune response [141].

Type I IFN effectively induces the family of tripartite motif (TRIM) proteins that also restrict HIV-1 replication. TRIM5a from rhesus macaque (RhTRIM5a) blocks the early replication steps of HIV-1 and other retroviruses [142] by targeting the incoming viral capsid (CA) protein for degradation. Anti-HIV-1 activity has also been reported for TRIM1 that also targets the CA protein at an early stage of pre-reverse transcription. TRIM19 was suggested to affect trafficking of viral proteins [143], and TRIM22 blocked the release of HIV-1 Gag particles [144].

It was shown that Type I IFN inhibits both acute and chronic retroviral (MuLV) infection and that the inhibition was at the posttranslational level affecting virus assembly and release [145]. HIV-1 replication was found to be also sensitive to IFN inhibition, and the block was mapped to the late stages of HIV-1 replication [146]. IFNα induced antiretroviral activities also include a decrease in proviral DNA accumulation [147], restriction of viral entry and viral nucleic acid synthesis [148], and in macrophages Type I IFN restricts HIV-1 replication during the initial stages of infection [149].

At least three ISGs were shown to affect HIV-1 assembly and release. ISG15, a ubiquitin-like protein inhibits HIV-1 infection [150] by interfering with the ubiquitination of HIV-1 Gag, blocking the interaction of TSG101 with Gag and consequently inhibiting the endosomal trafficking pathway, used by HIV-1 virions to exit cells [103]. The ISG 15 block is not specific for HIV-1, but also affects release of the Ebola virus particles [104]. TRIM22 blocks the intracellular trafficking of the viral structural protein Gag to the surface of the cell. The antiviral activity of TRIM22 is dependent on the E3 ligase activity of the RING-containing region of TRIM22 [144]. Finally, the cellular protein BST-2, also known as tetherin, has been shown to be a very effective inhibitor of HIV-1 release [138]. This protein is expressed constitutively in DC and B cells, but can be also effectively induced by Type I IFN in other cell types. Unlike the trapped retroviral virions in IFN treated cells, which have decreased infectivity, the HIV-1 virions retained on the cell surface by tetherin are infectious. The tetherin mediated inhibition is not specific but inhibits release of a large group of retroviruses, filoviruses and herpes viruses [151,152].

Altogether these results indicate that HIV-1 can induce a strong innate immune response, associated with type I IFN mediated induction of several ISGs with a profound anti HIV-1 activity. Therefore the absence of the innate antiviral control of HIV-1 replication has been somewhat puzzling. One reason is that HIV-1, like the other RNA viruses, developed mechanisms by which it down-regulates the action of the antiviral ISG. HIV-1 encodes three small accessory proteins Vif, Vpr and Vpu, which circumvent the innate immune response by targeting the cellular antiviral proteins APOBEC3G and tetherin for ubiquitin dependent proteasome degradation. The expression of Virion infectivity factor, Vif, is essential for HIV-1 replication in primary T cells [137]. In the absence of Vif, APOBEC3G is incorporated into HIV-1 virions; thus Vif prevents packaging of APOBEC3G and its availability during the very early stages of HV-1 infection [153]. Virion associated viral protein R (Vpr) stimulates proviral transcription, and similar to SIV encoding Vpx, facilitates infection of macrophages. Both Vpr and Vif interact with cullin associated ubiquitin complex [137]. Vpr and Vif also target IRF-3 for degradation in T cells, which results in inhibition of the antiviral response and IFNβ synthesis [113]. Finally, the membrane protein, Vpu, is required for efficient release of virus particles. Vpu interacts with the trans membrane domain of tetherin and targets it for proteasomal or lyosomal degradation [154,155]. Virions of HIV-1 mutants lacking the *vpu* gene accumulate on the plasma membrane of infected cells and are not released to the medium. Vpu also interacts with CD4 and targets it for degradation by the cullin associated ubiquitin complex [156].

**Figure 1:**
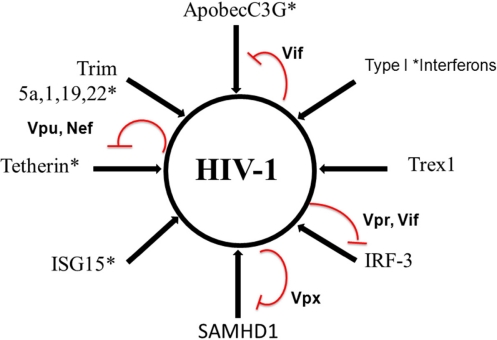
Schematic illustration of regulation of HIV-1 infection during innate antiviral response.

Other viral encoded functions may also counteract innate immunity. Interestingly, the negative factor Nef that has multiple functions, mostly in the modulation of adaptive immunity, can overlap Vpu and antagonize tetherin. In fact, primate SIV variants that lack Vpu use Nef to block the function of tetherin [157] (Figure 1).

The role of HIV-1 encoded protease was recently shown to play a role in the inhibition of Rig I mediated signaling induced by *in vitro* synthesized HIV-1 RNA. Ectopic expression of HIV-1 protease resulted in inhibition of IRF-3 phosphorylation and induction of ISG [158]. Since it is not clear whether ss HIV-1 RNA induces an interferon response in infected cells by the Rig I pathway, the *in vivo* relevance of this observation is yet to be confirmed.

In addition to virus encoded antiviral factors, several cellular proteins also contribute to down-regulation of the HIV-1 induced antiviral response. Thus the cellular DNAse Trex1 degrades the unintegrated proviral DNA in infected cells, thus preventing its detection by TLR9 or by the cytoplasmic DNA sensors. In TREX1 deficient cells HIV-1 DNA accumulates, which leads to induction of the IFN response [159]. Interestingly induction of IFN required the cellular adaptor STING and IRF-3, suggesting that proviral DNA is recognized by one of the cytoplamic DNA sensors, rather than by TLR9 [78]. Finally it was shown in SIV infected CNS macrophages, that IFNβ signaling is attenuated by induction of Suppressor of interferon signaling, SOCS3 [160]. Also the virus induced miRNA may modulate the antiviral response. *In vitro* studies have shown that several IFNβ induced miRNA including miR-26a, -34a, -145 targeted IFNB mRNA and attenuated the antiviral response [161]. Whether these miRNA also regulate the Type I IFN system in HIV-1 infected cells *in vivo* is yet to be determined.

## 7. Reflections and Future Considerations

Recent studies have clearly established that type I IFN has not only a critical role in the innate antiviral response but also in the pathogenicity of viral infection. Presently, some aspects of virus mediated induction of type I IFN and cytokine genes are understood at molecular levels, while others are still at a descriptive stage. The availability of genetically modified mice lacking essential components of the IFN signaling pathways confirmed that IFN is the earliest cellular defense against viral infection and a potent stimulant for the subsequent adaptive immune response. Rather unexpected was the observation that type I IFN induced large de-repression of the cellular genome stimulating expression of a large number of cellular genes, that are not only antiviral but are also involved in many different cellular functions. While it is easy to see the advantage generated by redundancy of antiviral proteins in their function against a wide variety of viruses, it is less evident why IFN evolved to control not only immune, but also many cellular functions. One has to wonder whether the multiplicity of IFN controlled genes reflect the number of cellular functions used by viruses for replication and survival. Important insights into viral pathogenicity have emerged from the observation than some of the nonstructural viral proteins target components of the IFN system, thereby decreasing or eliminating the antiviral response.

The innate antiviral response leads to expression of a large number of inflammatory cytokines in addition to interferon. The increased understanding of the cross talk of IFN pathway with the other inflammatory cytokines, illustrates an increasing degree of complexity in the mechanism of IFN action. This is well demonstrated in HIV-1 infection, where the early response to infection leads to a massive inflammatory response consisting of production of type I IFN, and many inflammatory cytokines. Thus while transient expression of type IFN may limit viral spread in the initial stages of infection, the cytokines stimulate HIV-1 infection either directly or by activation of immune cells. Since the ectopic expression of IFN inhibits HIV-1 replication in T cells [162], future studies will no doubt explore the means not only how to eliminate HIV-1 mimicry, but also how to generate the IFN response in the absence of inflammatory cytokines.
